# Integrating MRI radiomics and deep learning for predicting pathologic complete response to neoadjuvant chemotherapy in breast cancer: a multicenter study

**DOI:** 10.1186/s12880-026-02578-8

**Published:** 2026-07-14

**Authors:** Min Wei, Zhenyu Shu, Maohua Lyu, Yanting Liang, Yuguo Wei, Ying Wang, Guangying Zheng

**Affiliations:** 1 Cancer Center, Department of Ultrasound Medicine, Zhejiang Provincial People’s Hospital, Affiliated People’s Hospital, Hangzhou Medical College, Hangzhou, Zhejiang China; 2 Cancer Center, Department of Radiology, Zhejiang Provincial People’s Hospital, Affiliated People’s Hospital, Hangzhou Medical College, Hangzhou, Zhejiang China; 3 Department of Ultrasound，Sichuan Provincial People’s Hospital, Chengdu, China; 4 Department of Radiology, Guangdong Provincial People’s Hospital (Guangdong Academy of Medical Sciences), Southern Medical University, Guangzhou, 510080 China; 5 Advanced Analytics, GE Healthcare, Hangzhou, China; 6 Department of Medical Ultrasonics, The First Affiliated Hospital of Guangzhou Medical University, 151 Yanjiang West Road, Guangzhou, 510120 China

**Keywords:** Deep learning, Radiomics, Machine learning, Breast cancer, Neoadjuvant chemotherapy

## Abstract

**Objective:**

Develop a multimodal fusion model combining MRI radiomics and deep learning (DL) to predict pathologic complete response (pCR) in breast cancer patients post-neoadjuvant chemotherapy (NACT).

**Methods:**

Patients with locally advanced breast cancer from two centers (Center 1: 404 training, Center 2: 174 testing) were enrolled. Pretreatment MRI radiomic features and DL-derived features were extracted, selected via Minimum Redundancy Maximum Relevance and Least Absolute Shrinkage and Selection Operator, and integrated with clinical risk factors into a joint multimodal fusion model using XGBoost. The model was validated internally on an independent test cohort and externally on the Duke Breast Imaging Dataset (284 patients) to assess generalizability across diverse populations and imaging protocols.

**Results:**

The fusion model achieved AUCs of 0.935 (training), 0.916 (testing), and 0.83 (external validation), surpassing standalone radiomics and DL models. External validation confirmed robustness to institutional variability, with strong calibration and clinical utility.

**Conclusion:**

The XGBoost-integrated radiomics-DL-clinical model accurately predicts pCR, validated across independent cohorts, and may optimize personalized treatment strategies.

**Supplementary information:**

The online version contains supplementary material available at https://doi.org/10.1186/s12880-026-02578-8.

## Introduction

Breast cancer is one of the most common malignant tumors among women globally. According to global cancer statistics, the incidence of breast cancer continues to rise, becoming one of the leading causes of cancer-related deaths [1]. Neoadjuvant chemotherapy (NACT), a preoperative treatment strategy, has been widely applied in the treatment of breast cancer patients, with pathological complete response (pCR) being one of the key criteria for assessing the efficacy of NACT. pCR is closely associated with long-term survival in patients [2]. Therefore, early prediction of pCR status holds significant clinical importance in guiding personalized treatment. However, patients show significant individual differences in their response to NACT, with some patients being ineffective to treatment or even experiencing adverse reactions [3]. As a result, accurately predicting whether a patient will achieve pCR status early in the treatment process has become a critical issue in clinical decision-making.

Currently, preoperative assessment of the efficacy of neoadjuvant therapy for breast cancer mainly relies on traditional imaging methods, especially magnetic resonance imaging (MRI). Among these, dynamic contrast-enhanced MRI (DCE-MRI), with its excellent soft tissue contrast and ability to provide rich vascular and physiological information, has been widely used for preoperative evaluation of breast cancer [4, 5]. Although DE-MRI provides important information during diagnosis, traditional imaging analysis methods typically rely on the subjective judgment of radiologists, which may be influenced by differences in diagnostic experience and skills, leading to issues such as poor reproducibility and low diagnostic accuracy [6]. Additionally, while quantitative parameters derived from contrast-enhanced MRI and other pulse sequences (such as Diffusion Weighted Imaging, DWI) have been shown to be helpful in predicting pCR [7], the predictive ability of single imaging parameters is limited and cannot comprehensively reflect the tumor’s heterogeneity and its response mechanisms to treatment [8]. Therefore, the limitations of traditional imaging analysis methods in predicting the efficacy of NACT in breast cancer have prompted the exploration of more efficient methods.

Radiomics, an emerging technology, extracts a large number of high-dimensional quantitative features from medical images to capture deep information such as tumor morphology, texture, and intensity distribution, making it an important tool for addressing this problem [9]. Compared to traditional imaging analysis methods, radiomics can extract more characteristics that represent the biological properties of tumors from imaging data, providing more comprehensive and detailed information for predicting treatment response. However, due to the lack of direct biological interpretation of radiomic features and their poor stability across different datasets, the reliability of prediction models and their clinical applicability have been challenging [10]. With the development of machine learning (ML) and deep learning (DL) technologies, the predictive ability of radiomics has been significantly enhanced [11]. Existing studies have shown that machine learning models based on MRI radiomic features can effectively predict the pCR status of breast cancer patients [12]. On the other hand, DL as a branch of machine learning, has the ability to automatically extract features and perform highly nonlinear modeling, which can further improve the accuracy and generalizability of prediction models [13]. DL has achieved notable success in breast cancer diagnosis and treatment response prediction, demonstrating promising prospects [14, 15]. However, there is currently a lack of systematic studies exploring the combined effect and advantages of radiomics, ML, and DL for pCR prediction.

This study aims to construct an efficient, accurate, and highly interpretable model for predicting pCR in patient with breast cancer, based on the joint application of MRI radiomics, DL, and ML algorithms. This model will provide scientific support for clinical decision-making, enabling personalized treatment and improving patient survival rates.

## Materials and methods

### Patient information

This retrospective study was approved by the Institution Ethics Committee. Due to the retrospective nature of the study, the requirement for written informed consent was waived. In this study, clinical and imaging data from 578 patients across two medical centers were retrospectively collected between December 2017 and September 2024. All patients underwent MRI scans 1–2 weeks prior to receiving NACT. Inclusion Criteria: (a) biopsy-confirmed invasive breast cancer without distant metastasis; (b) completion of NACT with no prior treatments; (c) surgery performed after NACT, and pathological evaluation conducted postoperatively. Exclusion Criteria: (a) incomplete NACT; (b) no surgery performed or no postoperative pathology evaluation; (c) unavailable MRI data. Because the MRI scanners used at the two medical centers had different acquisition and reconstruction parameters, to validate the generalizability of the model, 404 patients from Medical Center 1 were included as the training cohort for model construction, while 174 patients from Medical Center 2 were used as the testing cohort to evaluate model performance. Additionally, this study included 284 patients from the Duke Breast Imaging Dataset (https://sites.duke.edu/mazurowski/resources) as an external validation cohort to assess model stability. This dataset contains DCE-MRI data of 922 patients with biopsy-proven invasive breast cancer, as well as related clinical, pathological and genomic information [16].

### Clinical and pathological data collection

Clinical and pathological data were extracted from patient medical records, including age, menstrual status, clinical TNM stage, tumor grade, molecular marker status, molecular subtype, and NACT regimen. Treatment regimens were determined by HER2 status in accordance with the National Comprehensive Cancer Network guidelines, HER2-negative patients received an anthracycline- and taxane-based regimen (four cycles of epirubicin/cyclophosphamide followed by four cycles of paclitaxel), whereas HER2-positive patients received a taxane plus anti-HER2 therapy (six cycles of docetaxel with trastuzumab). After completing NACT, all patients underwent breast surgery. The surgical specimens were assessed by breast pathologists from both hospitals using standard histopathological analysis. Tumor response was evaluated according to the Miller-Payne grading system [17], with grades 5 categorized as pCR, and the remainder as non-pCR, as detailed in the supplementary materials.

### Image segmentation and preprocessing

All breast MRI scans were performed on a 3.0T MRI scanner at a local hospital, the supplementary materials provide details of the imaging protocol and parameters. For each patient, tumor regions were manually delineated on DCE-MRI images using ITK-SNAP software. The third phase exhibiting the most prominent enhancement was used for segmentation. Volumetric ROIs were drawn manually along the tumor contours on all consecutive slices covering the whole tumor, excluding areas of necrosis, calcification, or hemorrhage. All segmentations were independently performed by two experienced radiologists (9 and 13 years of experience in breast cancer diagnosis), who were blinded to the clinical and pathological information. To ensure robustness in feature extraction, image standardization was performed prior to feature extraction, including resampling the images to a voxel size of 1 × 1 × 1 mm^3^ and standardizing the gray levels to 32 bins to eliminate the impact of different scanner parameters on feature extraction, as detailed in the supplementary materials. To further mitigate inter-site variability in radiomic features arising from differences in MRI scanner types and acquisition protocols across the three cohorts, ComBat harmonization was subsequently applied to the extracted radiomic feature set. ComBat employs an empirical Bayes framework to estimate and remove additive and multiplicative batch effects attributable to site- or scanner-level differences, while preserving biologically meaningful variance. This step ensured that the radiomic features used for model construction were robust to inter-scanner variability and comparable across all three cohorts.

### Radiomics model construction

The PyRadiomics platform was used to extract radiomic features related to pCR from the volumes of interest (VOIs) [18]. The extracted features included original shape features, first-order statistical features, texture features and filter-based features, totaling 1132 features. To ensure consistency and robustness in the extracted features, the Spearman rank correlation coefficient (CC) between the feature sets extracted by the two radiologists was calculated. Features with a CC value greater than 0.8 were considered highly consistent and included in subsequent analysis [19]. Radiomic feature reproducibility was assessed using intra-observer and inter-observer ICCs; features with ICC > 0.75 were retained, as detailed in the supplementary materials. Additionally, to exclude non-reproducible, redundant or irrelevant features, feature dimensionality reduction was performed on the radiomic feature set extracted from the training cohort. First, the Minimum Redundancy Maximum Relevance (mRMR) method was used for dimensionality reduction, followed by Least Absolute Shrinkage and Selection Operator (LASSO) regression to identify the important features for model building. Finally, a radiomics model was constructed using logistic regression, and a radiomics score (rad-score) was computed for each patient to predict the likelihood of achieving pCR. The model’s accuracy was evaluated using the receiver operating characteristic (ROC) curve and area under the curve (AUC) on the training, testing and validation cohorts. Specific features and dimensionality reduction details are provided in the supplementary materials.

### Construction of the deep learning Model

In this study, we employed the ResNeXt50_32×4d deep learning architecture as the foundational model, utilizing pre-trained weights from PyTorch for transfer learning [20]. Based on the whole-tumor segmentation, the slice exhibiting the maximum tumor area was automatically identified. This slice, along with its immediate cranial and caudal adjacent slices, was extracted to form a three-channel pseudo-3D input. A 2.5D classification approach was implemented to predict pCR. To enhance model generalizability, data augmentation strategies were applied, including random horizontal/vertical flipping and rotations within a ± 15° range. The AdamW optimizer was adopted with a weight decay of 1e-3, batch size of 64, and initial learning rate of 0.001. the learning rate was dynamically adjusted using a cosine annealing schedule over 50 training epochs, with cross-entropy loss serving as the optimization objective. Model performance was evaluated through the AUC, sensitivity, and specificity. Calibration curves were further employed to validate the reliability of predicted probabilities. To enhance the spatial interpretability of the deep learning model, Gradient-weighted Class Activation Mapping (Grad-CAM) was applied to generate class activation heatmaps. Grad-CAM computes the gradient of the predicted class score with respect to the feature maps of the final convolutional layer, producing spatial heatmaps that highlight the tumor regions most influential to the model’s pCR prediction. Representative Grad-CAM heatmaps were overlaid on the corresponding DCE-MRI slices for both pCR and non-pCR cases to visually demonstrate the spatial focus of the deep learning model.

### Joint model construction and validation

**Fig. 1:**
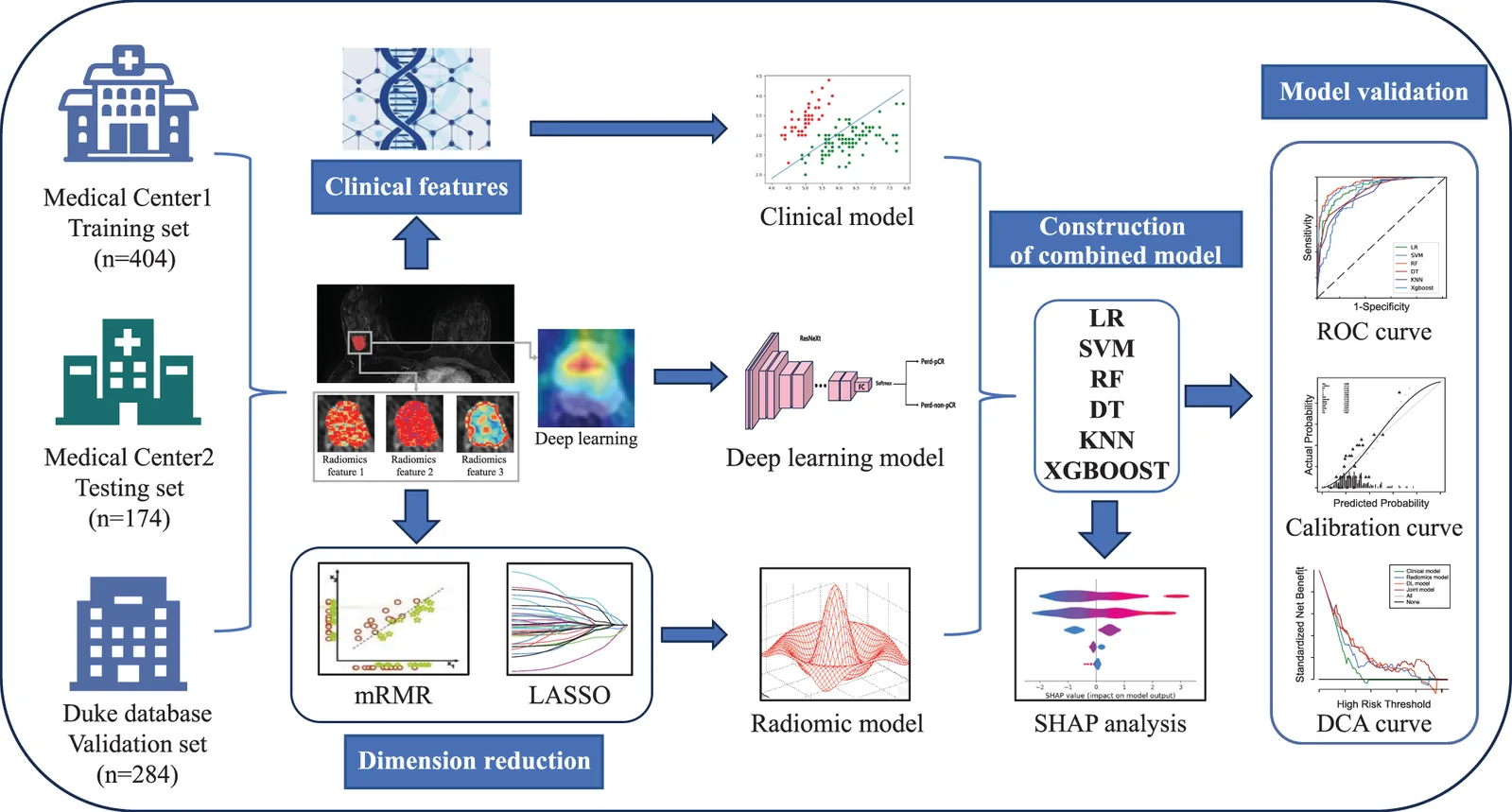
Research workflow for model construction and validation

For constructing the joint model, independent predictors were first selected from clinical features in the training cohort using multifactorial logistic regression, creating a clinical prediction model. Subsequently, the radiomics model and DL model were combined, and six machine learning algorithms were used to construct the joint model: logistic regression (LR), SVM, random forest (RF), decision tree (DT), K-nearest neighbors (KNN), and XGBoost. Each algorithm’s cross-validation process included two nested loops: the outer loop randomly split the training cohort into training and test subsets, repeated 200 times to evaluate classification performance, and the inner loop used 5-fold cross-validation to optimize the hyperparameters of the algorithm, which were automatically selected using the GridSearchCV toolkit [21]. Model performance was evaluated using ROC curves, and the machine learning algorithm with the highest AUC in the testing and validation cohorts was selected as the optimal algorithm. The Delong test was used to compare the performance differences between the joint model and other models, and calibration curves were used to check for model overfitting. The clinical net benefit of the joint model was assessed using decision curve analysis (DCA). In addition, to assess whether the joint model performed consistently across tumor biology, subgroup analyses stratified by molecular subtype were conducted in the training and internal testing cohorts, with the AUC, sensitivity, and specificity computed for each subgroup at the model’s predefined operating threshold. To address the black-box issue of machine learning models, a stratified interpretability framework was established. At the joint model level, SHapley Additive exPlanations (SHAP) analysis was applied to the best joint model, in which the DL predicted probability, radiomics score, and clinical features were treated as three modality-level input features. SHAP, grounded in cooperative game theory, calculates Shapley values to quantify the marginal contribution of each input feature to the model’s prediction output, enhancing transparency and interpretability at the fusion level. At the individual radiomics feature level, SHAP applied to the standalone logistic regression-based radiomics model to evaluate the contribution of each of the selected radiomic features to pCR prediction. At the deep learning level, Grad-CAM was employed for spatial interpretability. This tiered approach ensures methodologically appropriate and clinically meaningful interpretability across all model components. The research workflow is shown in Fig. 1. More detailed information about ML and SHAP analysis is provided in the supplementary materials.

### Statistical analysis

Statistical analysis was performed using SPSS version 24.0, MedCalc version 11.2, R version 4.3.1, and Python version 3.7.3. The normality of quantitative data was assessed using the Kolmogorov-Smirnov test. For normally distributed data, independent sample t-tests were used to compare differences between groups, and the data were expressed as Mean ± SD. For non-normally distributed data, the Mann-Whitney U test was used, with results expressed as median (interquartile range, M[IQR]). Categorical data were presented as frequency and percentage (n, %), and intergroup comparisons were made using the chi-square test (χ^2^ test). Two-sided tests with a *p*-value < 0.05 were considered statistically significant. Complete software environment specifications, including all library version numbers and hardware configuration details, are provided in the Supplementary Materials.

## Results

### Patient characteristics

In the training cohort, significant differences in all clinical characteristics were found between pCR and non-pCR patients (*p* < 0.05). In the testing cohort, significant differences were found for all clinical characteristics between pCR and non-pCR patients except for Menstruation, Clinical T-stage, Clinical Stage, and Age (*p* < 0.05). In the validation cohort, due to missing data for ER, Ki67, and Stage, these characteristics were excluded from the study. Among the remaining clinical features, HER2, Molecular Subtypes and Age showed significant differences between pCR and non-pCR patients (*p* < 0.05), while no significant differences were observed for the other characteristics (see Table 1 for details).

**Table 1:** Clinical characteristics of patients in the training, testing and validation cohorts

Cohort		Training cohort (*N* = 404)			Testing cohort (*N* = 174)			validation cohort (*N* = 284)		
Features	status	No-pCR (*N* = 255)	pCR (*N* = 149)	*p* value	No-pCR (*N* = 110)	pCR (*N* = 64)	*p* value	No-pCR (*N* = 219)	pCR (*N* = 65)	*p* value
Menstruation	Premenopausal	82 (32.2%)	66 (44.3%)	0.047	44 (40%)	17 (26.6%)	0.19	84 (38.4%)	34 (52.3%)	0.118
	Perimenopausal	115 (45.1%)	53 (35.6%)		43 (39.1%)	32 (50%)		98 (44.7%)	21 (32.3%)	
	Postmenopausal	58 (22.7%)	30 (20.1%)		23 (20.9%)	15 (23.4%)		37 (16.9%)	10 (15.4%)	
ER	Negative	65 (25.5%)	83 (55.7%)	<0.001	33 (30%)	42 (65.6%)	<0.001	NA	NA	NA
	Positive	190 (74.5%)	66 (44.3%)		77 (70%)	22 (34.4%)		NA	NA	
PR	Negative	81 (31.8%)	89 (59.7%)	<0.001	40 (36.4%)	45 (70.3%)	<0.001	NA	NA	NA
	Positive	174 (68.2%)	60 (40.3%)		70 (63.6%)	19 (29.7%)		NA	NA	
Her2	Negative	170 (66.7%)	57 (38.3%)	<0.001	70 (63.6%)	17 (26.6%)	<0.001	172 (78.5%)	38 (58.5%)	0.002
	Positive	85 (33.3%)	92 (61.7%)		40 (36.4%)	47 (73.4%)		47 (21.5%)	27 (41.5%)	
Ki.67	Negative	67 (26.3%)	23 (15.4%)	0.016	24 (21.8%)	3 (4.7%)	0.005	NA	NA	NA
	Positive	188 (73.7%)	126 (84.6%)		86 (78.2%)	61 (95.3%)		NA	NA	
Molecular subtypes	HR + and HER2–	136 (53.3%)	26 (17.4%)	<0.001	54 (49.1%)	4 (6.2%)	<0.001	148 (67.6%)	32 (49.2%)	0.011
	HER2+	85 (33.3%)	92 (61.7%)		40 (36.4%)	47 (73.4%)		16 (7.3%)	11 (16.9%)	
	Triple-negative	34 (13.3%)	31 (20.8%)		16 (14.5%)	13 (20.3%)		55 (25.1%)	22 (33.8%)	
Clinical T stage	T1–T2	191 (74.9%)	126 (84.6%)	0.031	82 (74.5%)	52 (81.2%)	0.408	170 (77.6%)	52(80%)	0.743
	T3–T4	64 (25.1%)	23 (15.4%)		28 (25.5%)	12 (18.8%)		49(22.4%)	13 (20%)	
Clinical N stage	No-metastasis	70 (27.5%)	60 (40.3%)	0.008	25 (22.7%)	31 (48.4%)	0.001	92 (42%)	27 (41.5%)	0.331
	metastasis	185 (72.5%)	89 (59.7%)		85 (77.3%)	33 (51.6%)		127 (58%)	38 (58.5%)	
Clinical.stage	I	18 (7.1%)	11 (7.4%)	0.004	3 (2.7%)	5 (7.8%)	0.264	NA	NA	NA
	II	146 (57.3%)	108 (72.5%)		70 (63.6%)	41 (64.1%)		NA	NA	
	III	91 (35.7%)	30 (20.1%)		37 (33.6%)	18 (28.1%)		NA	NA	
Age	Median (IQR)	51.00 (44.00 to 59.00)	49.00 (41.00 to 55.00)	0.027	49.00 (43.00 to 55.00)	52.50 (45.00 to 56.00)	0.163	49.00 (42.50 to 59.00)	47.00 (38.00 to 55.00)	0.032

Note. NA = not available; not available for all DUKE study participants

### Radiomics and deep learning model development and accuracy

**Fig. 2:**
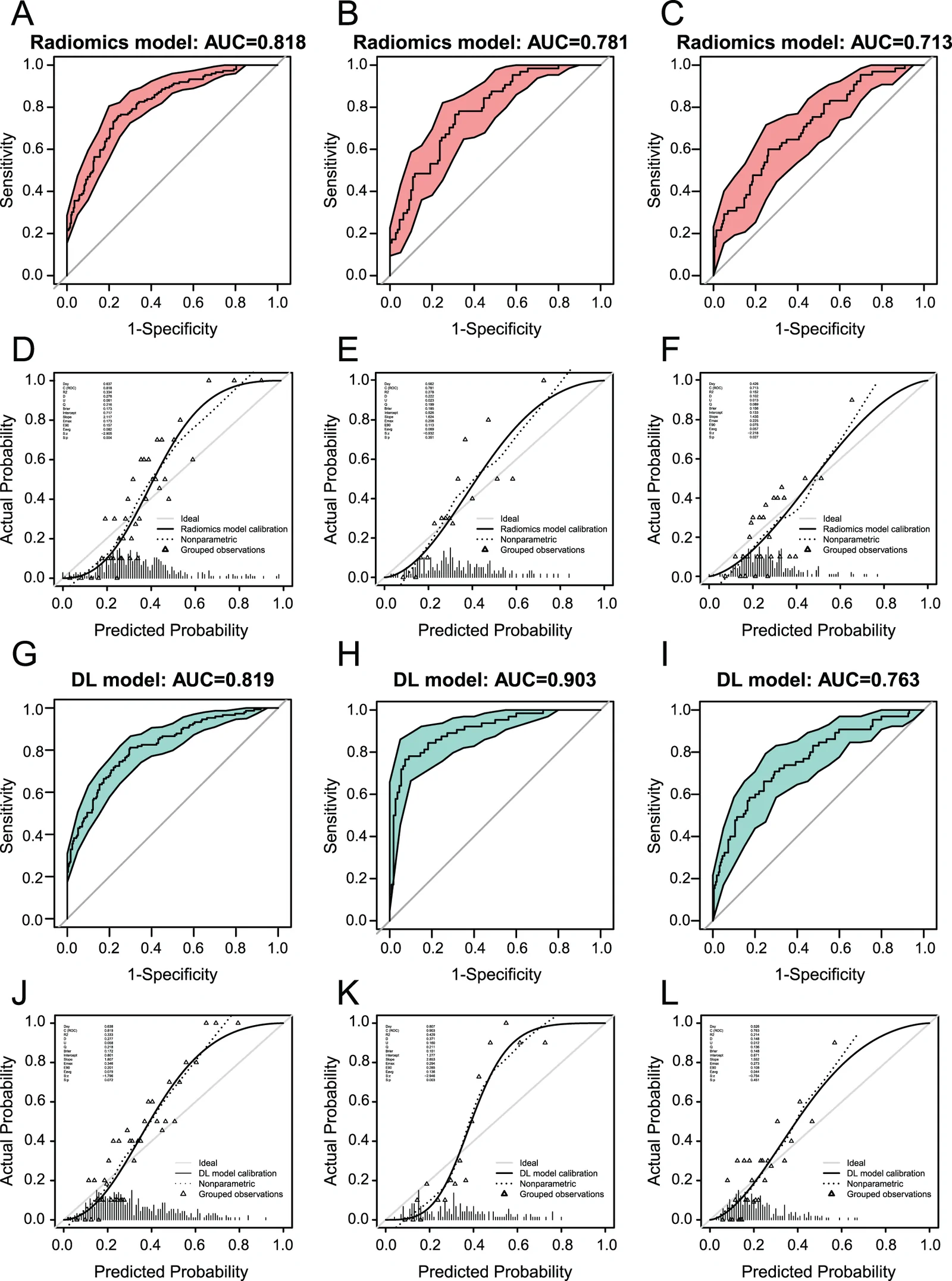
Panels **A**-**C** show the diagnostic performance of the radiomics model in the training, testing and validation cohorts. Panels **D**-**F** display the calibration curves of the radiomics model in the training, testing and validation cohorts. Panels **G**-**I** show the diagnostic performance of the deep learning model in the training, testing and validation cohorts. Panels **J**-**L** display the calibration curves of the deep learning model in the three cohorts

PCA demonstrated that prior to harmonization, the three cohorts formed distinctly separated clusters in the principal component space (Dim1 = 39.6%, Dim2 = 14.1%). Following ComBat harmonization, the three cohorts exhibited markedly improved distributional overlap (Dim1 = 41.8%, Dim2 = 16.5%) (Supplementary Figure S1). After feature dimensionality reduction, 13 features were retained for the construction of the radiomics model, including 2 original features, 1 square feature and 10 wavelet features. The details of the reduced features are provided in the supplementary materials. The radiomics model constructed from these features demonstrated good diagnostic performance in the training, testing, and validation cohorts, with AUCs of 0.810, 0.781, and 0.713, respectively. Additionally, the DL model based on ResNeXt50 showed superior diagnostic performance in all three cohorts, with AUCs of 0.818, 0.903, and 0.763, respectively. Calibration curves indicated that neither the radiomics model nor the DL model exhibited overfitting in the training, testing or validation cohorts (Fig. 2).

### Joint model construction and validation

**Fig. 3:**
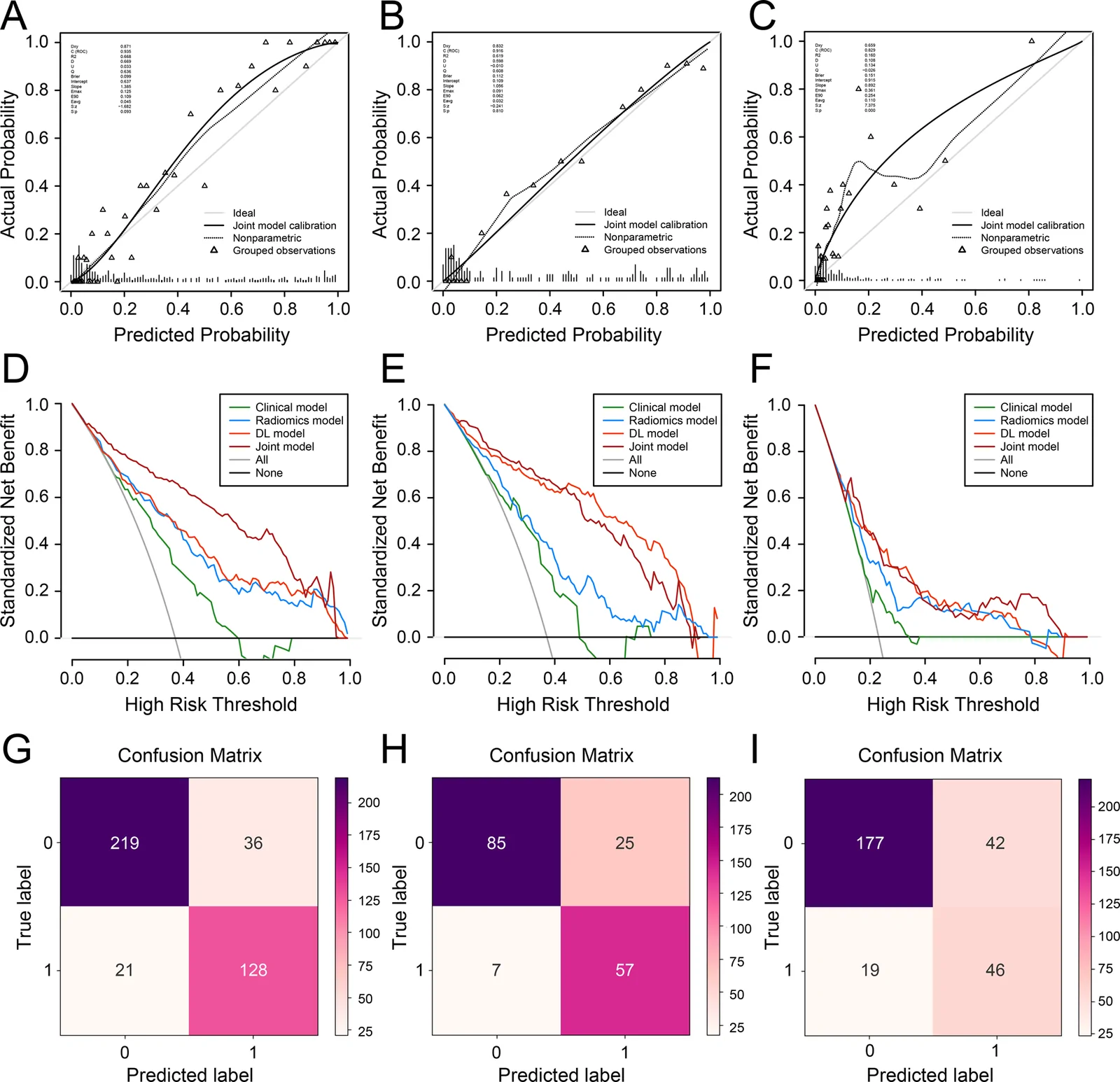
Panels **A**-**C** display the calibration curves of the joint model in the training, testing and validation cohorts. Panels **D**-**F** show the DCA benefit curves for the joint model, deep learning model, radiomics model and clinical model in the training, testing and validation cohorts, illustrating that the joint model provides the highest clinical net benefit across all three cohorts. Panels **G**-**I** display the confusion matrices for the joint model across the three cohorts, confirming the model’s excellent classification performance

**Fig. 4:**
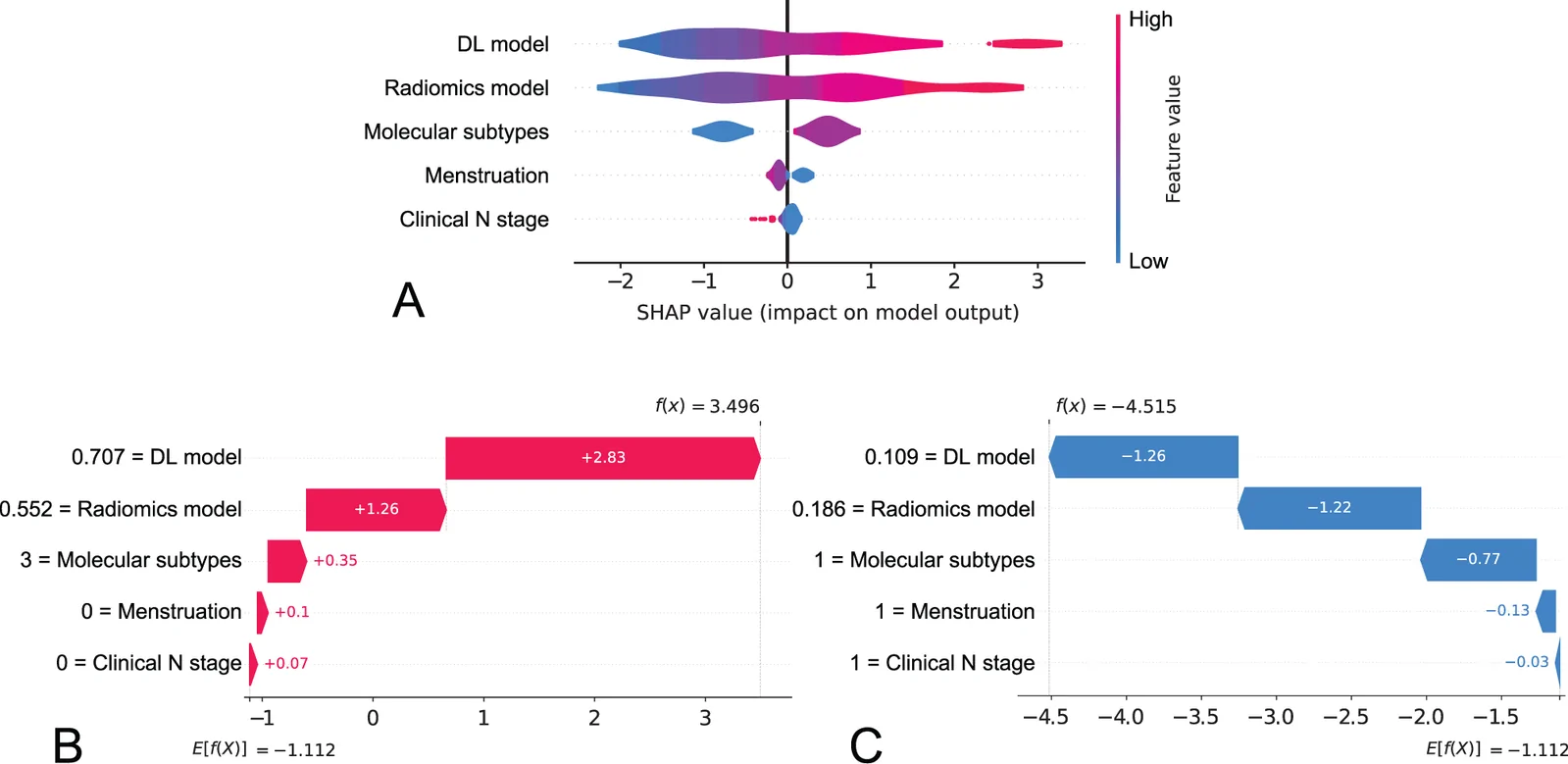
Panel **A** shows the distribution of SHAP values for each feature in the joint model, representing the impact of each feature on the model output. Color indicates feature value: red represents higher values, and blue represents lower values. The horizontal axis represents the distribution of SHAP values, and the vertical axis shows the contribution of each feature, with each point representing a sample. Points to the left of the vertical center line have SHAP values less than zero, indicating a negative impact on the prediction, while points to the right have SHAP values greater than zero, indicating a positive impact. Panels **B** and **C** show the SHAP waterfall plots for a pCR and a non-pCR patient, respectively. Each prediction starts from a baseline, which is the average Shapley value of all predictive features. The length of each colored arrow represents the contribution of the feature to the prediction, with red arrows indicating positive contributions and blue arrows indicating negative contributions. In the figure, E[f(x)] represents the baseline predicted probability, and f(x) represents the final predicted probability for the given input

**Fig. 5:**
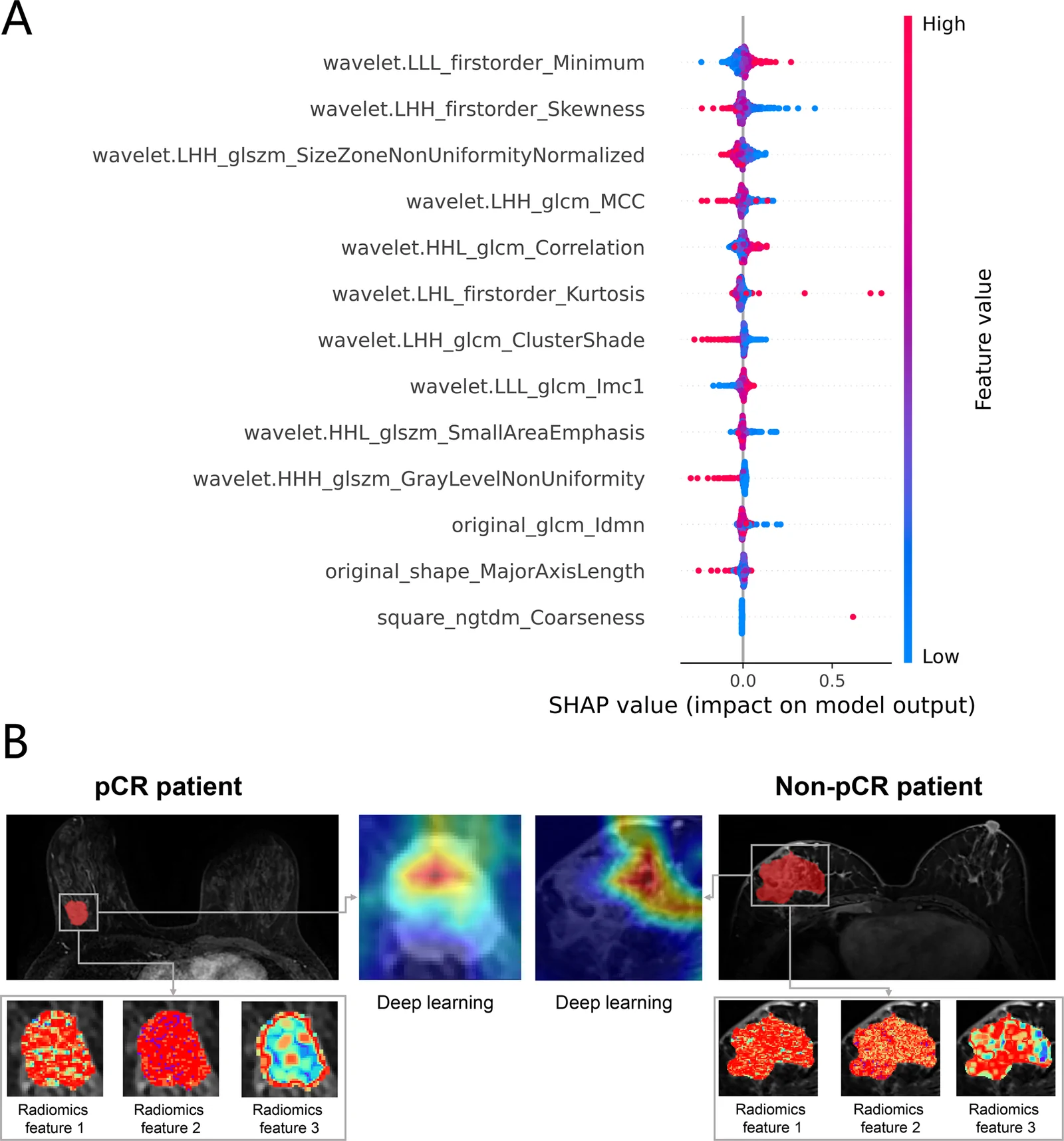
Panel **A** shows the SHAP beeswarm plot for the standalone radiomics logistic regression model, displaying the contribution of each of the 13 selected radiomic features to pCR prediction, ranked by mean absolute SHAP value. Panel **B** shows the integrated spatial interpretability visualization for a pCR-positive case (left) and a pCR-negative case (right). For each case, three visualization layers are presented: (i) The original DCE-MRI slice with the manually delineated tumor ROI (red overlay); (ii) Grad-CAM shows warmer colors (red/yellow) indicate spatial regions of higher importance to the DL model’s pCR prediction, the pCR-positive case shows concentrated activation within the tumor core and peri-tumoral enhancement zone, whereas the pCR-negative case exhibits comparatively diffuse activation; (iii) voxel-wise spatial distribution maps of the top three selected radiomic features within the tumor ROI, displayed as pseudo-color overlays in which color intensity reflects the local feature value at each voxel position, enabling visualization of intra-tumoral texture and intensity heterogeneity captured by the radiomic feature extraction pipeline

Multivariate logistic regression analysis identified Menstruation, Molecular Subtypes, and Clinical N-stage as independent predictors of pCR (see Table 2). The clinical model constructed based on these features showed AUCs of 0.725, 0.716, and 0.591 in the training, testing and validation cohorts, respectively. The joint model was constructed by combining the clinical model with the radiomics and DL models using six ML algorithms. ROC curve analysis revealed that, regardless of whether in the training, testing or validation cohort, the joint model based on the XGBoost algorithm exhibited the best diagnostic performance, with AUCs of 0.935, 0.916, and 0.83, respectively (see supplementary materials, Table S2). Subgroup analyses of the joint model stratified by molecular subtype were performed in the training and internal testing cohorts and are summarized in Table S3. In the internal testing cohort, the joint model achieved AUCs of 0.884 (HER2-positive), 0.934 (HER2-negative) and 0.894 (triple-negative), with consistent performance in the training cohort (0.928, 0.927 and 0.888, respectively), indicating strong and broadly stable discrimination across molecular subtypes. Subtype-stratified performance was not assessed in the external validation cohort because ER and PR data were incomplete in the Duke dataset, precluding reliable molecular-subtype stratification (Table S3). Furthermore, Delong tests showed that, except for no significant difference in diagnostic performance between the joint model and the DL model in the testing cohort, the joint model outperformed the other models in diagnostic performance (*p* < 0.05) (see Table 3). Calibration curves further indicated that the joint model did not exhibit overfitting in any of the three cohorts. The Decision Curve Analysis (DCA) showed that the joint model had higher clinical net benefit than the other models. Confusion matrix analysis also validated the excellent classification performance of the joint model (Fig. 3). SHAP analysis of the joint model revealed that the DL model predicted probability contributed the highest SHAP value among all input features, followed by the radiomics score and clinical features (Fig. 4A). Waterfall plots for two representative cases illustrated individual feature-level contributions, and the pCR patient showed a final predicted score of f (x) = 2.637, with the radiomics score (0.661) and DL predicted probability (0.443) both exerting positive SHAP contributions, while the non-pCR patient yielded f(x) = −5.062, with both imaging-derived features contributing negatively to the prediction (Fig. 4B and C). At the individual radiomic feature level, and the beeswarm plot of all 13 selected radiomic features ranked by mean absolute SHAP value showed that wavelet-derived features dominated the prediction, (Fig. 5A). Grad-CAM heatmaps showed spatially concentrated activation within the tumor ROI across representative cases, with activation patterns visually distinguishable between pCR-positive and pCR-negative patients (Fig. 5B).

**Table 2:** Identification of independent predictors for pCR based on multivariate logistic regression

Variables	Univariate logistic regression		Multivariate logistic regression	
	OR（95%CI）	*P***-value**	OR（95%CI）	*P***-value**
Age	0.98 (0.96–1.00)	0.020	NA	NA
Menstruation	0.57 (0.36–0.91)	0.017	0.45 (0.25–0.82)	0.009
Molecular.subtypes	5.66 (3.39–9.45)	<0.001	5.09 (2.62–9.88)	<0.001
Clinical T stage	0.54 (0.32–0.92)	0.024	NA	NA
Clinical N stage	0.30 (0.15–0.61)	<0.001	0.27 (0.13–0.58)	<0.001

Note. NA = not available

**Table 3:** Comparison of diagnostic performance of various models in training, testing, and validation sets

Group	Model	AUC (95% CI)	Sensitivity	Specificity	Delong_p
Training cohort	Clinical model	0.725 (0.674–0.773)	0.826	0.553	<0.001
	Radiomics model	0.810 (0.769–0.853)	0.752	0.753	<0.001
	Deep learning model	0.818 (0.773–0.859)	0.812	0.702	<0.001
	Combined model	0.935 (0.909–0.958)	0.866	0.859	NA
Testing cohort	Clinical model	0.716 (0.636–0.787)	0.891	0.491	<0.001
	Radiomics model	0.781 (0.712–0.846)	0.656	0.755	<0.001
	Deep learning model	0.903 (0.851–0.946)	0.922	0.627	0.570
	Combined model	0.916 (0.872–0.953)	0.891	0.773	NA
Validation cohort	Clinical model	0.591 (0.507–0.675)	0.462	0.726	<0.001
	Radiomics model	0.713 (0.637–0.780)	0.323	0.863	0.005
	Deep learning model	0.763 (0.691–0.833)	0.492	0.863	0.017
	Combined model	0.830 (0.778–0.884)	0.723	0.804	NA

## Discussion

This study proposed and validated a joint model integrating radiomics and DL models, constructed using the XGBoost algorithm, for personalized prediction of pCR in breast cancer patients after NACT. The results demonstrated that the joint model can accurately identify pCR patients, especially with good stability across multi-center datasets, which highlights the model’s cross-center applicability. This approach effectively showcases the synergistic effects between different models, and by incorporating explainability analysis, it enhances the transparency and generalizability of the model, thereby providing clinicians with additional evidence to support treatment decisions.

In recent years, radiomics has been widely applied in the diagnosis, grading and prognosis of tumors [22]. However, many early radiomics studies, though performing well in specific cohorts, are often limited by the singularity of feature extraction methods or the limited size of training datasets, leading to poor generalizability and stability of the models [23]. Moreover, while ML models excel in processing large-scale image data, their “black-box” nature makes it difficult to clarify the relationship between predicted results and specific clinical indicators [24]. To address these challenges, this study utilized SHAP analysis to provide a detailed contribution analysis of the features in the joint model, clearly demonstrating the specific impact of each feature on the prediction, particularly the weight of the DL prediction values. In fact, DL has been shown to provide insights into individual treatment responses, and our study further supports this [25]. By providing feature contribution explanations, this analysis fills the gap of insufficient transparency in machine learning models for clinical applications.

Compared to currently reported multi-factor clinical models or single radiomics models, the joint model performs better in terms of performance metrics. Previous studies have shown that combining clinical models with imaging features can improve pCR prediction, but most of them are limited to simple linear regression or low-dimensional feature spaces, which are insufficient for addressing the complex heterogeneity of patients [7]. For example, studies based on molecular subtypes or pathological staging of breast cancer patients, while validating the importance of single features in predicting pCR, fail to comprehensively reflect the complex pathological characteristics of patients [26]. Additionally, human experts often face subjectivity and variability when assessing the effectiveness of NACT, making it difficult to consistently and accurately identify changes in the tumor microenvironment induced by treatment [27]. This study, by introducing high-dimensional features extracted via DL and combining them with key radiomics features reduced through dimensionality reduction, along with clinical features, further enhanced the accuracy of the predictive model across multiple datasets. Compared to traditional clinical models, the DL and radiomics models constructed in this study exhibited higher AUC values in predicting pCR status, validating the feasibility of using deep learning techniques with MRI images [28]. This result suggests that DL and radiomics models may be able to capture advanced abstract features related to tumor microenvironment changes induced by NACT that are not visible to the human eye.

One notable strength of this study lies in the model’s generalizability, as evidenced by its validation using multi-center datasets, which demonstrated that the joint model’s performance in the external validation cohort closely mirrored its performance in the testing cohort, thereby underscoring its robustness and wide applicability. In contrast, models validated solely on single-center datasets are often susceptible to overfitting or biases stemming from specific population characteristics [29]. Furthermore, the multi-information fusion strategy employed in this study significantly enhanced the model’s performance, an advancement of paramount importance for clinical implementation [30]. DCA further substantiated the superior clinical utility of the joint model, revealing consistently higher net benefits across various threshold ranges compared to alternative models, a finding that can be largely attributed to the implementation of the XGBoost algorithm. Unlike traditional methods such as SVM or random forests, XGBoost excels in managing high-dimensional, nonlinear relationships while offering robust and consistent validation across diverse datasets [31]. These observations align with the findings of Kai et al., whose research similarly demonstrated the reliability of XGBoost-based models in predicting pCR in breast cancer patients undergoing NACT [32].

It is also worth noting that the relative contribution of each modality differed across cohorts. In the internal testing cohort, the combined model and the standalone DL model did not differ significantly, indicating that the DL representation already captured most of the available predictive signal within this cohort. On the independent external cohort, however, the combined model significantly outperformed the standalone DL model, suggesting that the harmonized radiomic and clinical components contribute complementary, site-stabilized information that becomes most valuable under distribution shift. Taken together, these findings indicate that multimodal fusion functions primarily as a robustness mechanism under distribution shift: it provides limited additional benefit when a single modality is already near-optimal on in-distribution data, but yields clear and significant gains in generalization to previously unseen institutions.

To contextualize these results against human expert performance, we note that a meta-analysis of DCE-MRI for evaluating pathological response reported a pooled sensitivity of 0.80 (95% CI 0.70–0.88) and specificity of 0.84 (95% CI 0.79–0.88) [33], with reported performance varying considerably across molecular subtypes [34]. Against this benchmark, our joint model achieved an AUC of 0.916 in the internal testing cohort and 0.830 in the external validation cohort with balanced sensitivity and specificity, suggesting that the multimodal fusion model provides discrimination at least comparable to conventional radiologist MRI assessment, while offering a fully reproducible, operator-independent prediction obtained before chemotherapy. In direct numerical terms, the joint model sensitivity of 0.891 in the internal testing cohort exceeded the pooled radiologist sensitivity of 0.80 by approximately 9% points, and its AUC of 0.916 surpassed the discrimination implied by that pooled operating point, with this performance achieved entirely from pretreatment imaging and free of the inter-reader variability that limits visual MRI assessment. A prospective multi-reader study nonetheless remains a valuable direction for future work.

Despite the significant progress made in this study, there are still some limitations. First, although the study includes a relatively large multi-center dataset, further validation with more samples and research centers is needed to enhance the robustness and generalizability of the model. Second, the current deep learning pipeline adopted a 2.5D strategy that extracts only the three largest cross-sectional slices of the tumor ROI, which does not fully exploit the volumetric information contained across all tumor-bearing slices. Future work may therefore explore multi-slice embedding aggregation strategies to more comprehensively characterize the entire tumor volume from MRI and potentially further improve pCR prediction performance. Third, although the stratified interpretability framework established in this study provides transparency at multiple levels, the DL predicted probability itself represents a high-level abstract feature derived from a neural network and cannot be directly mapped to specific biological or morphological tumor characteristics. This remains an inherent interpretability limitation of the current fusion framework. With the continuous development of big data and artificial intelligence technologies, future studies may explore breast MRI-specific foundation models as alternative deep learning backbones, as well as automated feature selection and real-time model update strategies, to further enhance feature representation, prediction performance, and clinical utility. At last, the external validation cohort lacked key clinical biomarkers, necessitating application of the joint model with incomplete clinical inputs. However, given that the clinical feature model alone yielded the lowest predictive performance among all component models, and the primarily attributable to its imaging-derived components. Future validation in cohorts with complete multimodal data remains warranted. In addition, ComBat harmonization was applied only to the radiomic feature set; no image-level domain adaptation was performed for the deep learning inputs, and the cross-site robustness of the deep learning component was therefore not formally quantified, and future work should incorporate dedicated image-level domain-adaptation techniques and formally evaluate the deep learning model’s robustness across sites.

This study demonstrates the potential of a joint model combining radiomics and ML in predicting pCR status in breast cancer. By integrating tumor imaging and relevant clinical features, the model provides a more comprehensive evaluation of patients’ conditions after NACT, offering a non-invasive, personalized prediction method for breast cancer patients.

## Electronic supplementary material

Below is the link to the electronic supplementary material.

Supplementary Material 1

## Data Availability

The Duke Breast Imaging Dataset used for external validation in this study is publicly available at https://sites.duke.edu/mazurowski/resources. The clinical and imaging data from Center 1 and Center 2 used for model training and internal testing are not publicly available due to patient privacy restrictions, but may be available from the corresponding author upon reasonable request.

## Abbreviations

pCR: Pathologic complete response
NACT: Neoadjuvant chemotherapy
MRI: Magnetic resonance imaging
DCE-MRI: Dynamic contrast-enhanced MRI
DWI: Diffusion weighted imaging
DL: Deep learning
ML: Machine learning
ROI: Region of interest
VOI: Volume of interest
AUC: Area under the curve
ROC: Receiver operating characteristic
mRMR: Minimum Redundancy Maximum Relevance
LASSO: Least Absolute Shrinkage and Selection Operator
XGBoost: Extreme Gradient Boosting
SVM: Support vector machine
RF: Random forest
DT: Decision tree
KNN: K-nearest neighbors
LR: Logistic regression
DCA: Decision curve analysis
SHAP: SHapley Additive exPlanations
ER: Estrogen receptor
PR: Progesterone receptor
HER2: Human epidermal growth factor receptor 2
HR: Hormone receptor
TNM: Tumor-Node-Metastasis
IQR: Interquartile range
CC: Correlation coefficient
