# Supplementary material for: Integrating MRI radiomics and deep learning for predicting pathologic complete response to neoadjuvant chemotherapy in breast cancer: a multicenter study
Source: BMC Med Imaging. 2026 Jul 14;26:505. doi: 10.1186/s12880-026-02578-8 (PMC13647561; doi:10.1186/s12880-026-02578-8)
Supplement: Supplementary file 1 [file 12880_2026_2578_MOESM1_ESM.docx]

**Supplementary Materials**

**1.1 NAC Regimen and Criteria for pCR**

According to the National Comprehensive Cancer Network Guidelines(1), all patients received the standard six to eight cycles of NACT before surgery. HER-2 negative patients received 4 cycles of epirubicin and cyclophosphamide treatment, followed by 4 cycles of paclitaxel treatment. HER-2 positive patients received 6 cycles of docetaxel and trastuzumab treatment, and all patients underwent surgical treatment after NACT. The surgical specimens were fixed in 10% neutral buffered formalin and overnight processing in a standard tissue processor. Slides are cut at 5μm and dyed in an automatic dyeing system. Histopathological examinations and analyses are performed by pathologists with at least 12 years of experience in breast pathology who turn a blind eye to MRI data. Specimens were pathologically assessed using the Miller-Payne system(2). pCR was defined as the absence of residual invasive tumors (Miller-Payne grade 5, possible presence of residual ductal carcinoma in situ), the absence of lymph node infiltration in the ipsilateral sentinel lymph nodes or lymph nodes removed during axillary dissection (ypT0/isN0).

**1.2 Definition of** **Miller-Payne grade system**

Gread 1: No change or some alteration to individual malignant cells but no reduction in overall cellularit.

Gread 2: A minor loss of tumour cells but overall cellularity still high, and up to 30% loss.

Gread 3: Between an estimated 30% and 90% reduction in tumour cell.

Gread 4: A marked disappearance of tumour cells such that only small clusters or widely dispersed individual cells remain, and more than 90% loss of tumour cells.

Gread 5: No malignant cells identiﬁable in sections from the site of the tumour, only vascular fibroelastotic stroma remains often containing macrophages. However, ductal carcinoma in situ(DCIS) may be present.

**1.3 MRI acquisition**

All breast MRI scans were performed in a local hospital on a 3.0 T MRI scanner ((Signa HDxt, GE Healthcare, USA). The routine sequences included T2-weighted imaging(T2WI), diffusion-weighted imaging(DWI) and dynamic contrast-enhanced(DCE).

T2WI: repetition time/echo time= 3800/80 ms, field of view= 340×340 mm, matrix= 384×224, section thickness= 5 mm,Slice Gap=1mm,flip angle= 90°.

DWI: repetition time/echo time= 6030/68ms, field of view= 360×360 mm, matrix= 108×129, section thickness= 5 mm,Slice Gap=1mm,flip angle= 90°.

DCE: repetition time/echo time= 5.65/2.46ms, field of view= 360×360 mm, matrix= 384×384, section thickness= 2.5 mm,Slice Gap=0.5mm,flip angle= 15°.

**1.4 Image preprocessing**

Image preprocessing including resample, intensity normalization and gray-level discretization were performed with the Pyradiomics program. To be more specific, image preprocessing was performed by resampling the images with a resolution of 1×1×1 mm3 through the linear interpolation method and by discretizing and normalizing the image gray level to order 32. Extracted texture features were standardized, which removed the unit limits of the data of each feature and converted it into a dimensionless pure value. This allowed the indexes of different units or orders to be compared and weighted. We used a z-score normalization to make the image intensities fit a standard normal distribution with $\mu\text{=0}$ and $\sigma\text{=1}$, where $\mu$ is the mean value of the images, and $\sigma$ is the standard deviation. The normalized values (also called z-scores) of the image intensities (x) were calculated as follows:

$$z=\frac{x-\mu}{\sigma}$$

**1.5 Intraclass Correlation Coefficient (ICC)**

To evaluate the robustness of radiomic feature extraction to segmentation variability, both intra-observer and inter-observer agreements were assessed using the intraclass correlation coefficient (ICC). For intra-observer analysis, one radiologist re-segmented the randomly selected subset of 50 patients after a 4-week interval. For inter-observer analysis, the same subset was independently segmented by two radiologists.

ICC values for all radiomic features are shown in the histograms . Features with ICC > 0.75 were considered robust and retained for subsequent model construction, while those with ICC ≤ 0.75 were excluded. The distribution of ICC values was heavily skewed toward 1.00 for both intra-observer and inter-observer analyses, indicating excellent overall reproducibility of the radiomic features.

**1.6 ComBat Harmonization of Radiomic Features**

To address inter-site heterogeneity in radiomic features introduced by differences in MRI acquisition parameters across the three cohorts (Center 1, Center 2, and the Duke external validation cohort), ComBat harmonization was applied after radiomic feature extraction and prior to feature selection. ComBat is an empirical Bayes method originally developed for genomic batch effect correction and has been widely validated for harmonizing radiomic features in multicenter imaging studies. The method models the observed feature value *Y_ijg_* for feature g, site *_i_*, and sample *_j_* as:

*Y_ijg_ = α_g_ + X_ij_β_g_ + γ_ig_ + δ_ig_εi_jg_*

where *α_g_* denotes the overall feature mean, *X_ij_β_g_* captures the biological covariate effects (e.g., pCR status), *γ_ig_* represents the additive batch effect, *δ_ig_* the multiplicative batch effect, and *εi_jg_* the error term. Empirical Bayes shrinkage is used to estimate *γ_ig_* and *δ_ig_*, with the pCR label included as a protected covariate to ensure that harmonization removes only technical variation without distorting the biological signal of interest. The harmonized features were used in all subsequent feature selection and model construction steps.

**Figure S1.** PCA visualization of radiomic features before and after ComBat harmonization. Panel A shows the PCA plot of all radiomic features prior to harmonization, with data points colored by cohort (Center 1, Center 2, Duke). The clear clustering by cohort indicates the presence of site-related batch effects. Panel B shows the corresponding PCA plot after ComBat harmonization, demonstrating substantially reduced inter-cohort distributional discrepancies and improved overlap across the three cohorts, confirming the effectiveness of the harmonization procedure.

**1.7** **Details of Dimension reduction**

First, the minimum redundancy maximum relevance (mRMR) algorithm was used to extract robust features from the dataset. The aim of the the minimum redundancy process ensured that the selected features had minimal redundancy among the other features. At the same time, maximum relevance procedure was to select features having the maximum correlation with the sports subtype，and we selected features with correlation coefficients greater than 0.8 and 0.1 as high correlation and low redundancy features, respectively. Then, the mRMR method was used to obtain an optimal feature set with a high correlation and low redundancy.

At last, dimensionality reduction of the selected features was performed using the least absolute shrinkage and selection operator (LASSO) method. LASSO is a powerful algorithm for regression analysis with high dimensional predictors. The LASSO algorithm shrinks some coefficients and reduces others to exactly 0 via an absolute constraint. Thus, LASSO is an outstanding method for feature selection as it retains good features using both a subset selection and ridge regression. In this study, LASSO selected 13 nonzero coefficients, and detailed of features and dimension reduction can be found in Table S1 and Figure S1-S2.

**Table S1. The information of radiomics features**

| Feature Groups (N) | Feature names | Feature Groups (N) | Feature names |
| --- | --- | --- | --- |
|  | firstorder_10Percentile |  | glszm_GrayLevelNonUniformity |
|  | firstorder_90Percentile |  | glszm_GrayLevelNonUniformityNormalized |
|  | firstorder_Energy |  | glszm_GrayLevelVariance |
|  | firstorder_Entropy |  | glszm_HighGrayLevelZoneEmphasis |
|  | firstorder_InterquartileRange |  | glszm_LargeAreaEmphasis |
|  | firstorder_Kurtosis |  | glszm_LargeAreaHighGrayLevelEmphasis |
|  | firstorder_Maximum |  | glszm_LargeAreaLowGrayLevelEmphasis |
| First-order features (N = 18) | firstorder_MeanAbsoluteDeviation | GLSZM texture features | glszm_LowGrayLevelZoneEmphasis |
|  | firstorder_Mean | (N = 16) | glszm_SizeZoneNonUniformity |
|  | firstorder_Median |  | glszm_SizeZoneNonUniformityNormalized |
|  | firstorder_Minimum |  | glszm_SmallAreaEmphasis |
|  | firstorder_Range |  | glszm_SmallAreaHighGrayLevelEmphasis |
|  | firstorder_RobustMeanAbsoluteDeviation |  | glszm_SmallAreaLowGrayLevelEmphasis |
|  | firstorder_RootMeanSquared |  | glszm_ZoneEntropy |
|  | firstorder_Skewness |  | glszm_ZonePercentage |
|  | firstorder_TotalEnergy |  | glszm_ZoneVariance |
|  | firstorder_Uniformity |  |  |
|  | glrlm_GrayLevelNonUniformity |  | glcm_Autocorrelation |
|  | glrlm_GrayLevelNonUniformityNormalized |  | glcm_ClusterProminence |
|  | glrlm_GrayLevelVariance |  | glcm_ClusterShade |
|  | glrlm_HighGrayLevelRunEmphasis |  | glcm_ClusterTendency |
|  | glrlm_LongRunEmphasis |  | glcm_Contrast |
| GLRLM texture features | glrlm_LongRunHighGrayLevelEmphasis | GLCM texture features | glcm_Correlation |
| (N = 16) | glrlm_LongRunLowGrayLevelEmphasis | (N = 24) | glcm_DifferenceAverage |
|  | glrlm_LowGrayLevelRunEmphasis |  | glcm_DifferenceEntropy |
|  | glrlm_RunEntropy |  | glcm_DifferenceVariance |
|  | glrlm_RunLengthNonUniformity |  | glcm_Id |
|  | glrlm_RunLengthNonUniformityNormalized |  | glcm_Idm |
|  | glrlm_RunPercentage_T2 |  | glcm_Idmn |
|  | glrlm_RunVariance |  | glcm_Idn |
|  | glrlm_ShortRunEmphasis |  | glcm_Imc1 |
|  | glrlm_ShortRunHighGrayLevelEmphasis |  | glcm_Imc2 |
|  | glrlm_ShortRunLowGrayLevelEmphasis |  | glcm_InverseVariance |
|  |  |  | glcm_JointAverage |
|  |  |  | glcm_JointEnergy |
|  |  |  | glcm_JointEntropy |
|  |  |  | glcm_MCC |
|  |  |  | glcm_MaximumProbability |
|  |  |  | glcm_SumAverage |
|  |  |  | glcm_SumEntropy |
|  |  |  | glcm_SumSquares |
|  | gldm_DependenceEntropy |  | ngtdm_Busyness |
|  | gldm_DependenceNonUniformity |  | ngtdm_Coarseness |
| GLDM texture features | gldm_DependenceNonUniformityNormalized | NGTDM texture features | ngtdm_Complexity |
| (N = 14) | gldm_DependenceVariance | (N = 5) | ngtdm_Contrast |
|  | gldm_GrayLevelNonUniformity |  | ngtdm_Strength |
|  | gldm_GrayLevelVariance |  |  |
|  | gldm_HighGrayLevelEmphasis |  |  |
|  | gldm_LargeDependenceEmphasis |  |  |
|  | gldm_LargeDependenceHighGrayLevelEmphasis |  |  |
|  | gldm_LargeDependenceLowGrayLevelEmphasis |  |  |
|  | gldm_LowGrayLevelEmphasis |  |  |
|  | gldm_SmallDependenceEmphasis |  |  |
|  | gldm_SmallDependenceHighGrayLevelEmphasis |  |  |
|  | gldm_SmallDependenceLowGrayLevelEmphasis |  |  |
| Los features (N = 186) | Log-sigma-1.0，2.0_* (N =186) |  |  |

Note: GLCM, Gray-level co-occurrence matrices; GLRLM, Gray-level run length matrix; GLSZM, Gray-level size zone matrix; GLDM, Gray-level dependence matrix. *The abbreviated representation of feature types

**Figure S1**. Figures A and B respectively show the selection of hyperparameters in the LASSO dimensionality reduction process.

**Figure S2**. After dimensionality reduction, the remaining 13 features are sorted, and the red bars represent the weight values of the features.

**1.8 Machine Learning Details**

In our study, we used 6 machine-learning classifiers (Logistic regression, Bayes, XGBoost, K-Nearest neighbors, Random forest and Support vector machines). Nested cross-validation process was used for each type of machine learning modeling (**Figure S3**). At last, we focused on and validated the XGBoost model because of its better performance. Detailed details can be found in Table S2.

**Table S2.** Comparison of diagnostic performance of 6 machine learning algorithms in the three sets

| Group | Model | AUC (95% CI) | Accuracy | Sensitivity | Specificity | Delong_p |
| --- | --- | --- | --- | --- | --- | --- |
| Training set | LR | 0.907 (0.875-0.936) | 0.842 | 0.805 | 0.863 | <0.001 |
|  | SVM | 0.849 (0.811-0.885) | 0.755 | 0.893 | 0.675 | <0.001 |
|  | RF | 0.947 (0.925-0.967) | 0.894 | 0.846 | 0.922 | 0.0059 |
|  | DT | 0.879 (0.844-0.911) | 0.824 | 0.685 | 0.906 | <0.001 |
|  | KNN | 0.863 (0.827-0.898) | 0.79 | 0.725 | 0.827 | <0.001 |
|  | Xgboost | 0.935 (0.909-0.958) | 0.861 | 0.866 | 0.859 | / |
| Testing set | LR | 0.914 (0.869-0.953) | 0.833 | 0.875 | 0.809 | 0.8167 |
|  | SVM | 0.836 (0.772-0.890) | 0.747 | 0.906 | 0.655 | 0.0007 |
|  | RF | 0.910 (0.863-0.949) | 0.851 | 0.891 | 0.827 | 0.3717 |
|  | DT | 0.891 (0.840-0.937) | 0.793 | 0.672 | 0.864 | 0.1023 |
|  | KNN | 0.855 (0.794-0.910) | 0.793 | 0.812 | 0.782 | 0.0117 |
|  | Xgboost | 0.916 (0.872-0.953) | 0.816 | 0.891 | 0.773 | / |
| Validation set | LR | 0.806 (0.749-0.867) | 0.718 | 0.785 | 0.699 | 0.0766 |
|  | SVM | 0.702 (0.633-0.777) | 0.673 | 0.615 | 0.689 | <0.001 |
|  | RF | 0.817 (0.762-0.876) | 0.789 | 0.646 | 0.831 | 0.2496 |
|  | DT | 0.800 (0.737-0.860) | 0.746 | 0.769 | 0.74 | 0.2189 |
|  | KNN | 0.768 (0.705-0.833) | 0.768 | 0.508 | 0.845 | 0.0363 |
|  | Xgboost | 0.830 (0.778-0.884) | 0.785 | 0.723 | 0.804 | / |

Note: LR, Logistic Regression.SVM, Support vector machine. RF, Random forest. DT, Decision tree. KNN, K-Nearest neighbor. XGBoost, eXtreme Gradient Boosting.

**Figure S3.** The cross validation process of machine learning algorithm based on training queue.

**eXtreme Gradient Boosting (XGBoost)**

When dealing with small and medium structured data, the decision tree algorithm is the best. Additionally, XGBoost is an integrated machine-learning algorithm based on decision trees, framed by Gradient Boost. To train the model, we should define the objective function to measure the fitting degree of the model to the training data. The salient characteristic of objective functions is that it consists of training loss and a regularization term. Moreover, the XGBoost technique optimizes both parts of the model. As a result, the XGBoost model provides a more accurate prediction model and effectively prevents overfitting.

**Decision tree (DT)**

The decision tree is a decision analysis method used to evaluate project risks and determine feasibility by calculating the probability that the expected net present value (NPV) is greater than or equal to zero, based on the known probabilities of various scenarios. It is a graphical method that intuitively applies probability analysis.

**Random forest (RF)**

Random forest is an ensemble machine learning method for classification and regression, which operated by constructing a large number of decision trees and outputting classes as a single tree (classification) or average prediction (regression) model. Random forest provides an improvement to bagging with a modification step of random sampling of predictors.

**Support vector machine (SVM)**

Support vector machine is a machine learning approach which is based on the structural risk minimization principle of statistics learning. It projected data into a multidimensional space and classified it with hyper-planes.

**K-Nearest neighbor (KNN)**

The k-Nearest Neighbor (KNN) classification algorithm is a theoretically mature method and one of the simplest machine learning algorithms. The idea of this method is that in the feature space, if most of the k nearest (i.e. the closest) samples near a sample belong to a particular category, then the sample also belongs to that category.

**Logistic regression** **(LR)**

Logistic regression analysis is a statistical method which is used to analyze a data set in which one or more independent variables that determine the outcome. The outcome was measured by a dichotomous variable, in which there were only two possible outcomes.

**1.81 Hyperparameter Optimization and Model Configuration**

All machine learning classifiers were optimized using grid search with nested cross-validation. The outer loop employed 5-fold cross-validation for unbiased performance estimation, while the inner loop used 5-fold cross-validation for hyperparameter selection. The optimal hyperparameter configurations identified for each model are summarized as follows.

For the Support Vector Machine (SVM), the optimal parameters were: regularization parameter C = 0.001, kernel function = linear, and gamma = 0.01. For the Random Forest (RF), the optimal configuration was: number of estimators = 25, maximum tree depth = 4, and maximum number of features = 4. For the Decision Tree (DT), the optimal parameters were: splitting criterion = Gini impurity, splitter = random, maximum features = 2, and maximum depth = 5. For the K-Nearest Neighbor (KNN) classifier, the optimal settings were: number of neighbors = 18, search algorithm = ball_tree, leaf size = 1, and distance weighting = uniform. For the XGBoost model, the optimal hyperparameters were: number of estimators = 24, maximum tree depth = 2, column subsampling ratio = 0.4, row subsampling ratio = 0.7, and minimum child weight = 1.

**1.9** **Visualization of the prediction SHAP (SHapley additive explanation)**

With the increasing arithmetic power of machine learning, models become increasingly complex. An XGBoost model often contains tens or hundreds of boosting trees, which highly reduces the interpretability of the model. To understand the inherent causality of model complexity, SHapley Additive exPlanations (SHAP) was used to analyze the relationship between the features and output in the XGBoost ‘black box’. SHAP analysis evaluated the SHAP value of each feature of the training sample, which represented the sensitivity of that feature to changes in model output. By linearly decomposing the prediction result into the effect of each feature, the importance of features could be calculated and the role of different features in the model could be visualized. And explains the model’s prediction as the sum of the Shapley values of each input feature:

$g\left( x’ \right)=\varphi_{0}+\sum_{M}^{j=1} \varphi_{j}$

where g (x’) is the value of the model, Φ0 is the constant that explains the model (i.e., the predicted mean of all training samples), and Φj is the imputed value (Shapley value) of each feature.

**2 Computational Environment and Software Specification**

All model training, feature extraction, and statistical analyses were performed on a single workstation equipped with an AMD Ryzen 7 7700 8-core processor (base clock 3.80 GHz), 32.0 GB system RAM, and an NVIDIA GeForce RTX 5060 Ti GPU (16 GB VRAM) with CUDA support for deep learning acceleration.

All machine learning, radiomics, and deep learning analyses were implemented in Python (version 3.7.3). Radiomic feature extraction was performed using PyRadiomics (version 3.0.1). Deep learning model construction and training were carried out with PyTorch (version 1.13.1, CUDA 11.7). The joint predictive model was built using XGBoost (version 1.7.3), with feature selection and cross-validation implemented via scikit-learn (version 1.0.2). Model interpretability was assessed using SHAP (version 0.41.0). Data processing and management were conducted with NumPy (version 1.21.6) and Pandas (version 1.3.5). Batch effect correction was performed using the R package sva (version 3.56.0) in R (version 4.3.1). Additional statistical analyses were completed using SPSS (version 24.0) and MedCalc (version 11.2).

**Table S3.** Diagnostic performance of the joint model within molecular-subtype subgroups.

| **Molecular subtype** | **Cohort** | **AUC (95% CI)** | **Sensitivity** | **Specificity** | **Accuracy** |
| --- | --- | --- | --- | --- | --- |
| **HER2-positive** | Training | 0.928 (0.890–0.963) | 0.913 | 0.800 | 0.859 |
|  | Internal testing | **0.884 (0.799–0.951)** | 0.915 | 0.700 | 0.816 |
|  | External validation | 0.879 (0.791–0.947) | 0.778 | 0.809 | 0.797 |
| **HER2-negative** | Training | 0.927 (0.883–0.963) | 0.789 | 0.888 | 0.863 |
|  | Internal testing | **0.934 (0.876–0.980)** | 0.824 | 0.814 | 0.816 |
|  | External validation | 0.830 (0.778–0.884) | 0.723 | 0.804 | 0.785 |
| **Triple-negative** | Training | 0.888 (0.798–0.962) | 0.781 | 0.765 | 0.773 |
|  | Internal testing | **0.894 (0.765–0.986)** | 0.923 | 0.625 | 0.759 |
|  | External validation | NA | NA | NA | NA |

Note: AUC = area under the receiver operating characteristic curve; CI = confidence interval; NA = not available. Molecular-subtype subgroup analysis was not performed (reported as NA) in the external validation (Duke) cohort because estrogen receptor (ER) and progesterone receptor (PR) data were incomplete in that dataset, which precluded reliable molecular-subtype stratification.

**Reference**

1.	Goetz MP, Gradishar WJ, Anderson BO, et al. NCCN Guidelines Insights: Breast Cancer, Version 3.2018. J Natl Compr Canc Netw 2019;17(2):118-126.

2.	Ogston KN, Miller ID, Payne S, et al. A new histological grading system to assess response of breast cancers to primary chemotherapy: prognostic significance and survival. Breast 2003;12(5):320-327.
